# The impact of stigma on medication adherence among patients with multimorbidity: mediation analysis of medication literacy

**DOI:** 10.3389/fpubh.2026.1782369

**Published:** 2026-04-30

**Authors:** Chunyan Feng, Xiaojing Zhang, Menglan Shen, Rongsong Tang, Chunyan Su

**Affiliations:** 1 Department of Nursing, Peking University Third Hospital, Beijing, China; 2 Department of General Medicine, Peking University Third Hospital, Beijing, China; 3 Department of Endocrine, Peking University Third Hospital, Beijing, China; 4 Department of Nephrology, Peking University Third Hospital, Beijing, China

**Keywords:** medication literacy, medication adherence, stigma, multimorbidity, mediation analysis

## Abstract

**Purpose:**

The relationship among stigma, medication literacy and medication adherence in patients with multimorbidity is limited. This study was to explore the relationships among medication literacy, stigma, and medication adherence.

**Patients and methods:**

This cross-sectional study adopted a convenient sampling method to survey 221 patients with multimorbidity who attended the general practice department of a tertiary hospital in Beijing, China, from March to October 2023. A self-designed questionnaire was used to collect the patients' sociodemographic data; the Chinese version of the Medication Literacy Scale (C-MLS), Stigma Scale for Chronic Illness (SSCI), and Morisky Medication Adherence Scale were applied to assess their medication literacy, stigma, and medication adherence, respectively. As medication adherence score, medication literacy, and stigma were all continuous variables, analysis of variance (ANOVA) was employed for univariate analysis. To examine the associations among these three variables, pairwise correlation analysis was first performed, followed by mediation analysis to explore their intrinsic relationships. Model fit was evaluated using the Root Mean Square Error of Approximation (RMSEA), Comparative Fit Index (CFI), Normed Fit Index (NFI), and Tucker-Lewis Index (TLI); RMSEA < 0.08 and CFI, NFI, TLI all > 0.90 indicated a good model fit.

**Results:**

A total of 221 patients with multimorbidity were included: 123 (55.66%) with 2–3 concurrent chronic diseases, 72 (32.58%) with 4–5, and 26 (11.76%) with ≥6 chronic conditions. Stigma was negatively associated with medication adherence (*r* = −0.259, *p* < 0.001) and medication literacy (*r* = −0.293, *p* < 0.001), while medication adherence was positively associated with medication literacy (*r* = 0.188, *p* < 0.01). Mediation analysis indicated a well-fitted model (χ^2^/df = 1.12, CFI = 0.997, TLI = 0.992, RMSEA = 0.024, SRMR = 0.041). The indirect association between stigma and medication adherence via medication literacy was significant [β = −0.045, SE = 0.022, 95% CI = (−0.095, −0.001)].

**Conclusions:**

Medication literacy, stigma, and medication adherence were closely correlated, and medication literacy may serve as an intermediate variable in the relationship between stigma and medication adherence.

## Introduction

Multimorbidity is commonly defined as the co-occurrence of at least two chronic conditions in the same individual (1). The number of multimorbidity patients is gradually increasing, the prevalence of chronic disease comorbidities in China in recent years has ranged from 40%−70% (2). Compared to individuals with a single chronic condition (3), those patients with multimorbidity face a higher risk of premature mortality, more hospital admissions and longer hospital stays (4). Additionally, polypharmacy—when an individual takes five or more medications at the same time—is linked to adverse outcomes in older adults, including poor medication adherence, frailty, hospitalization, and even higher mortality (5, 6). Medication adherence refers to the extent to which a person's medication-taking behavior corresponds with agreed upon treatment recommendations from a healthcare provider (7). High adherence to medication can significantly reduce complications and mortality rates among patients with multimorbidity (8). Medication adherence refers to the consistent use of medications as prescribed over time. However, non-adherence remains a common issue among patients with multimorbidity (9). Additionally, current interventions have not yet achieved the desired outcomes (10). In a retrospective study of older adults with multimorbidity, the average medication adherence rate was 71%, and nearly two-thirds of participants had adherence rates below 80% (11). Therefore, exploring the possible influencing factors and the mechanism of medication nonadherence, and then providing targeted interventions are important to improve medication adherence in patients having multimorbidity.

The possible risk factors for medication nonadherence of patients with multimorbidity are complex, including disease related factors (comorbidities, symptom, disease stage and duration, among others), treatment related factors (mainly medication regimen) and patients related factors (12). Psychosocial issues, one of patients' related factors, may also affect patients' medication adherence. Patients with multimorbidity and polypharmacy may experience guilt, anxiety, depression, and even suicidal behaviors due to financial strain, challenges in daily life, and insufficient social support (13). Perceptual/sensory impairment is a significant contributor to reduced medication adherence in older patients. Specifically, the perception of adverse drug reactions and overall cognitive function are independent predictors of poor medication adherence among older hypertensive patients (14). Studies have indicated patients with chronic diseases across various systems mild to moderate levels of stigma (15), which refers to the internalized sense of shame experienced by patients who feel labeled, marginalized, discriminated against, isolated, and misunderstood due to their illness condition (16). Patients with multimorbidity require long-term rehabilitation and medication, resulting in heavy economic, physical and psychological burdens. Chronic comorbidities are characterized by long duration, high costs and poor prognosis, often forcing patients to stop working and leading to unstable income (17). Stigma can undermine patients' self-management abilities and confidence in their treatment, while also diminishing their motivation to adhere to medication (16, 18). Stigma may reduce adherence to insulin therapy and blood glucose monitoring among patients with diabetes (19). In addition, with increasing disease duration, the adherence of patients with chronic diseases decreases (20), and stigma increases (21). However, the specific mechanisms underlying the relationship between stigma and medication adherence in patients with multimorbidity remain unclear and warrant further study.

According to the Health Belief Model (HBM), stigma heightens patients' perceived impairment (e.g., fear of social judgment) while reducing perceived benefits of treatment (e.g., doubting therapeutic efficacy), thereby directly undermining medication adherence (22). On the contrary, health literacy could be effective in reducing perceived barriers by creating sufficient awareness and increasing perceived benefits, and self-efficacy (23). Medication literacy is a kind of health literacy, which defined as the ability to access, comprehend, and apply medication-related information for informed decision-making (24). It serves as an independent predictor of medication adherence (25). Furthermore, a study has demonstrated that external stigma affects medication adherence via medication literacy, whereas internal stigma exerts its influence through self-efficacy **i**n people living with human immunodeficiency virus (26). Higher medication literacy mitigates perceived barriers (e.g., clarifying drug interactions) and strengthens self-efficacy (e.g., confidence in self-management), both of which collectively promote medication adherence (22). However, stigma could erode this protective effect by reducing patients' motivation to engage with health information, as seen in multimorbidity populations (27). Recent research corroborates this pathway, which demonstrated that stigma was negatively associated with both medication literacy and adherence (19). Therefore, we hypothesized that stigma could influence medication adherence via medication literacy in patients with multimorbidity. A single-center cross-sectional study was implemented to explore the relationship among stigma, medication literacy and medication adherence in patients with multimorbidity.

## Material and methods

### Study design

This cross-sectional study employed convenience sampling to recruit patients with multimorbidity from the general medicine ward (where mainly provided treatment for patients having multiple chronic conditions) in Peking University Third Hospital. The study received approval from the Medical Ethical Committee of Peking University Third Hospital (IRB2022-109-02). All methods were applied in accordance with the relevant guidelines and regulations.

### Participants and data collection

The patients with multimorbidity were enrolled from the general medicine ward of the hospital from March, 1st to October, 31st in 2023. Participants should meet the following inclusion criteria: Diagnosis of at least two of the predefined chronic conditions (hypertension, diabetes, coronary heart disease, cerebrovascular disease, hyperlipidemia, chronic kidney disease, chronic obstructive pulmonary disease, or malignancy, among others); Prescribed at least one chronic disease medication for a minimum of 3 months prior to enrollment. Exclusion criteria included: severe cognitive or communication impairments (e.g., dementia, aphasia); active psychiatric disorders (e.g., schizophrenia, major depressive disorder) affecting study participation; end-stage organ failure; Terminal illnesses with life expectancy < 6 months.

The questionnaire was distributed through the online wenjuanxing platform. The primary nurses who had received training explained the content and purpose of the study to the patients, and patients filled the questionnaire using their own cellphones if they agreed to participate in the study; otherwise, they might exit the questionnaire interface. For individuals with visual/hearing/mild cognitive impairments, nurses helped to fill out questionnaires by asking them each item. The system was set to block incomplete submissions, requiring nurses to assist with unfinished mandatory questions. A total of 230 questionnaires were collected. After data cleaning, nine responses were excluded due to patterned responding (i.e., respondents provided identical or mechanically consistent answers across all items, indicating inattentive or insincere responses). Finally, 221 valid responses were included in the statistical analysis (valid response rate 96.1%). Informed consent was obtained from all subjects and/or their legal guardian(s).

### Sample

Kline (28) recommends a minimum sample size of 10–15 times the number of observed variables for structural equation models to ensure stable parameter estimates and model fit evaluation. There are 15 variables [11 demographic items, two stigma dimensions (self-stigma and enacted stigma), one medication literacy dimension, and one medication adherence dimension] in this study. The sample size should be 167–250 to account for 10% of the invalid-responders. Finally, the actual sample size was 230 (221 valid cases), which met the requirements for analysis.

### Research tools

#### Sociodemographic information questionnaire

After reviewed the previous studies (20, 29) a self-designed questionnaire was used to collect patients' sociodemographic data and the information of disease and medications, including age, gender, education levels, marital status, payment scheme, number of chronic diseases, the duration of disease (the time span from the onset of the main diagnosis identified during this hospitalization), perceptual and sensory disorders, hospitalizations, and kinds of medication.

Perceptual and sensory disorders are highly prevalent symptoms in chronic conditions, we assume that they may affect patients' medication adherence (30). We collected a variety of perceptual and sensory disturbance conditions, including hearing impairment, visual impairment, somatosensory issues (e.g., partial numbness in limbs), mobility challenges (e.g., balance disorders or reliance on assistive devices), and cognitive impairment (e.g., impaired orientation, memory, or computational skills). Patients were asked to report whether they had each of these perceptual or sensory impairments. Patients with any of these impairments were defined as having perceptual/sensory impairment, otherwise not.

#### Stigma scale for chronic illness (SSCI)

This scale was originally developed by RAO to measure stigma in patients with neurological diseases (31). The Chinese version of the scale (32) includes 13 items related to self-stigma and 11 items related to s enacted stigma. Self-stigma is more about the patient's own experience and feeling, such as feeling distant from others, worrying about being excluded, fearing others' attitudes, being saddened by appearance changes from the disease, and feeling embarrassed when speaking. Enacted stigma refers more to others' attitudes and behaviors toward patients, such as avoiding, being unfriendly, teasing, or looking away to avoid eye contact, among others. The five-point Likert scoring method was adopted, and the options “no,” “rarely,” “sometimes,” “often” and “always” were calculated as 1–5, respectively, with total scores ranging from 24 to 120. The higher the score, the greater the degree of stigma experienced by the patients. The Cronbach's α coefficient of the scale was 0.951, and both the self and the enacted stigma dimensions were 0.927 (32).

#### Chinese version of the medication literacy scale (C-MLS)

This scale was originally developed by Sauceda et al. (24) and is used mainly to measure the ability of patients to read, understand, calculate and address drug-related problems in the medical information environment. There are four cases provided in this scale: Case 1 is injection drug use for diabetic patients, Case 2 is drug use for children, Case 3 is drug use with similar effects of different drug names, and Case 4 is non-prescription drugs and prescription drugs with supplementary instructions. The questionnaire has 14 items scored with a dichotomous system (1 point for a correct answer, 0 for incorrect). The total score of 14 is the sum of all item scores. Higher scores indicate better patient medication literacy. A total score < 4 is considered poor drug literacy, 4–10 is moderate drug literacy, and >10 is good drug literacy. The Chinese version of the medication literacy assessment scale is a one-dimensional scale with a retest reliability of 0.885, a fractional half reliability of 0.840 and a Kuder–Richardson 20 (K-R 20) score of 0.820 (33).

#### Chinese version of the medication adherence scale

The Chinese version of the medication adherence scale had good reliability and validity (34), and was widely used in the study of medication adherence of patients with chronic diseases (35). The scale contains eight items, items 1–4,6 and 7 (whether you sometimes forget to take medication; whether you forgot to take medication in the past 2 weeks; reduce the dose or stop taking the medication, whether you stop taking the medicine sometimes; whether you give up treatment because of inconvenience) are scored 0 for “yes,” 1 for “no.” Item 5 (whether you took medicine yesterday) is scored reversely (1 for “yes,” 0 for “no”). Items 8 (how often do you forget to take medication) is scored 1.00 (never), 0.75 (occasionally), 0.50 (sometimes), 0.25 (often) and 0 points (always). The total score of the questionnaire is the sum of each item, ranging from 0 to 8 points. A higher score indicates better adherence. The Cronbach's α is 0.776, the convergent validity correlation coefficient is 0.878, and the interclass correlation coefficient (ICC) is 0.854.

### Statistical analysis

Statistical analyses were performed by SPSS 25.0 (IBM Corporation, Armonk, NY, USA) and Mplus 8.3 (Muthén & Muthén, Los Angeles, CA, USA). Categorical variables were described using frequency and proportion, and continuous variables were described as mean ± standard deviation or median (IQR) according to the normality status. Independent *t*-tests or one-way ANOVA was used to compare the means differences of normality variables, such as medication adherence and medication literacy, among different demographic groups. Mann-Whitney *U*-test or Kruskal-Wallis one-way ANOVA test were used to compare the difference of stigma level among different demographic groups. Paired rank sum tests were performed to compare the average scores of the two dimensions of stigma. Bivariate correlation was used to analyze the relationships between stigma, medication literacy, medication adherence, and the number of diseases. Spearman correlation was used for non-normally distributed data (presented as median and IQR), and Pearson correlation was used for normally distributed data. We conducted the Harman's Single Factor Test to check for any significant bias in the data.

A mediation model to examine pathways that medication literacy mediates stigma's effect on adherence was constructed by Mplus 8.3. We took two steps to test the mediation effect by using the structural equation model. The first step was to establish the structural equation model, the independent variables included self-stigma, enacted stigma, and stigma, while the dependent variable was medication adherence. As self-stigma and enacted stigma represent conceptually distinct constructs with satisfactory psychometric properties, they were concurrently incorporated into the regression model without substantial multicollinearity concerns. In the second step, based on Model 1, added the mediating variable of medication literacy, with the covariates of age, gender, number of diseases, and perceptual/sensory disorders. Establish Structural Equation Model 2 to test whether medication literacy mediates the effect of stigma on medication adherence. The following indices indicate that the model fits well (36): Root Mean Square Error of Approximation (RMSEA) < 0.08, Comparative Fit Index (CFI) >0.90, Normed Fit Index (NFI) >0.90, and Tucker-Lewis Index (TLI) >0.90. Quantified indirect effects was assessed via bias-corrected bootstrapping (5,000 resamples). Full mediation means stigma affects adherence exclusively through medication literacy. Partial mediation indicates stigma exerts both direct and indirect effects on adherence.

## Results

### Sociodemographic information of the participants

A total of 221 patients with multimorbidity were analyzed in this study. The average age of the participants was 65.75 ± 11.47 (23–89 years), with 124 males and 97 females (Table 1). Among the 221 patients, the most common comorbidities were hypertension (147 cases, 66.5%), followed by diabetes (110 cases, 49.8%), hyperlipidaemia (86 cases, 38.9%), chronic kidney diseases (86 cases, 38.9%), and cardiovascular diseases (61 cases, 27.6%). All of the patients had at least two chronic conditions, and 96 people (44.18%) had four or more chronic diseases (Table 1). Among 221 patients, 67.4% had no perceptual/sensory impairments, while visual impairments were most common in the remainder (13.1%, Table 1).

**Table 1:** Characteristics of the participants (*N* = 221).

Variable	Categories	
Gender, *n* (%)	Male	124 (56.1)
	Female	97 (43.9)
Age, *n* (%)	≤ 45	15 (6.8)
	46–64	72 (32.6)
	≥65	134 (60.6)
Chronic disease, *n* (%)	Hypertension	147 (66.5)
	Diabetes	110 (49.8)
	Coronary heart diseases	61 (27.6)
	Hyperlipidemia	86 (38.9)
	Chronic renal diseases	86 (38.9)
	Osteoporosis	24 (10.9)
	Tumor or cancer	19 (8.6)
	Digestive system diseases^*^	18 (8.1)
	Nervous system diseases^*^	14 (6.3)
	Respiratory diseases^*^	12 (5.4)
	Arthritis	9 (4.1)
Level of education, *n* (%)	Illiteracy	8 (3.6)
	Middle school	92 (41.6)
	High school	81 (36.7)
	Bachelor	32 (14.5)
	Postgraduate and above	8 (3.6)
Marital status, *n* (%)	No spouse	22 (10.0)
	Having spouse	199 (90.0)
Provider payment, *n* (%)	Medical insurance	173 (78.3)
	Free medical service	31 (14.0)
	New rural cooperative medical system	15 (6.8)
	Self-paying	2 (0.9)
Co-resident, *n* (%)	With family	201 (91.0)
	Live alone	20 (9.0)
Number of chronic diseases, *n* (%)	2–3	123 (55.7)
	4–5	72 (32.6)
	>6	26 (11.8)
Duration of disease, *n* (%)	< 1	6 (2.7)
	1–3	41 (18.6)
	4–9	44 (19.9)
	≥10	130 (58.8)
Perceptual/sensory disturbance, *n* (%)	Hearing disorder	31 (14)
	Visual impairment	29 (13.1)
	Paresthesia	8 (3.6)
	Mobility impaired	26 (11.8)
	Cognitive disorder	12 (5.4)
	None	149 (67.4)
Hospitalization times, *n* (%)	First	79 (35.8)
	Many times	142 (64.2)
Type of medications, *n*%	1–2	48 (21.7)
	3–5	93 (42.1)
	5–9	71 (32.1)
	≥10	9 (4.1)
Stigma^#^, score [M (IQR)]	Total score	26 (24,42.5)
	Self –stigma (general)	15 (13,26)
	Enacted stigma (general)	11 (11,15)
	Self-stigma (average)	1.15 (1.0,2.0)
	Enacted stigma (average)	1.00 (1.00,1.36)
Medication adherence, score (mean ± SD)		6.15 ± 1.95
Medication literacy, score (mean ± SD)		8.26 ± 3.60

^*^Digestive system diseases: chronic gastritis or chronic enteritis; nervous system diseases: mild cognitive impairment, cerebral infarction, cerebral hemorrhage; respiratory diseases: asthma, pneumonia, chronic obstructive pulmonary disease.

^#^Self-stigma (average) = self-stigma (general)/item numbers of self-stigma, enacted stigma (average) = enacted stigma (general)/item numbers of enacted stigma.

The unrotated exploratory factor analysis extracted 49 distinct factors, with the first factor accounting for 37.76% of the total variance (below the critical threshold of 40%) (37), indicating no substantial common method bias in the dataset.

### Medication literacy, stigma and medication adherence status of the participants

Medication adherence, and medication literacy were approximately normally distributed. The medication adherence levels were moderate (6.15 ± 1.95, Table 1). Patients with more than six comorbidities had the worst medication adherence (5.19 ± 2.20, *p* < 0.05, Table 2). In addition, patients with perceptual/sensory disturbance had poor medication adherence (5.57 ± 1.98, *p* < 0.05, Table 2). The mean medication literacy score of the 221 patients with multimorbidity was 8.26 ± 3.60. The medication literacy of 30 patients (13.6%) was poor (total score < 4), 94 (42.5%) patients were moderate (total score 4–10), and 97 (43.9%) were good (total score >10). Patients with the new rural cooperative medical system or self-payment had lower medication literacy than those in the other two groups (*p* < 0.05, Supplementary Table S1).

**Table 2:** Univariate analysis of medication adherence score of patients with multimorbidity (*n* = 221).

Variable	Categories	Medication adherence score			
		*N* (%)	Mean ±SD	*F*/*t*	*p*-Value
Age (years)	≤ 45	15 (6.79)	6.13 ± 1.94	0.016	0.984
	46–60	72 (32.58)	6.19 ± 1.90		
	>60	134 (60.63)	6.13 ± 1.99		
Gender	Male	124 (56.11)	6.07 ± 2.02	0.690	0.491
	Female	97 (43.89)	6.25 ± 1.87		
Level of education	Junior high school and below	100 (45.25)	6.40 ± 1.72	2.319	0.101
	High school	81 (36.65)	5.77 ± 2.26		
	Bachelor and above	40 (18.10)	6.27 ± 1.74		
Marital status	No spouse	22 (10.0)	5.56 ± 2.30	2.296	0.131
	Having spouse	199 (99.0)	6.22 ± 1.91		
Payment scheme	Medical insurance	173 (78.28)	6.17 ± 1.96	0.143	0.866
	Free medical service	31 (14.03)	6.15 ± 2.03		
	NCMS and self-paying	17 (7.69)	5.65 ± 1.74		
Co-resident	With family	201 (91.0)	6.15 ± 1.96	0.634	0.427
	Live alone	20 (9.0)	6.48 ± 1.45		
Number of chronic diseases	2–3	123 (55.66)	6.36 ± 1.85	3.997	0.020^*^
	4–5	72 (32.58)	6.13 ± 1.95		
	≥6	26 (11.76)	5.19 ± 2.20		
Duration of disease (years)	0–3	47 (21.27)	5.85 ± 2.20	1.442	0.239
	4–9	44 (19.91)	5.93 ± 2.03		
	≥10	130 (58.82)	6.34 ± 1.82		
Perceptual/sensory disturbance	Yes	72 (32.58)	5.57 ± 1.98	2.997	0.003
	No	149 (67.42)	6.41 ± 1.89		
Hospitalization times	First	79 (35.75)	6.26 ± 1.76	0.627	0.531
	Many times	142 (63.25)	6.09 ± 2.06		
Kinds of medications	1–2	48 (21.72)	5.86 ± 2.03	0.495	0.686
	3–5	93 (42.08)	6.29 ± 1.94		
	5–9	71 (32.13)	6.15 ± 1.92		
	≥10	9 (4.07)	6.13 ± 2.11		

NCMS, new rural cooperative medical system.

^*^Patients with more than six kinds of chronic disease had lower medication adherence than patients in other two groups (*p* < 0.05).

The total stigma score was 26 (24, 42.5), with self-stigma 15 (13, 26) and enacted stigma 11 (11, 15). The average score of self-stigma was higher than that of enacted stigma [1.15 (1.00, 2.00) vs. 1.00 (1.00, 1.36), *Z* = −3.794, *p* < 0.001]. Patients who were unmarried, having more than 6 chronic conditions, with perceptual or sensory disturbances, or having multiple hospitalizations had higher stigma scores than their counterparts (*p* < 0.05, Supplementary Table S2).

### Correlations among medication literacy, stigma and medication adherence

The bivariate correlation analysis results (Table 3) revealed that stigma was negatively associated with medication adherence (*r* = −0.259, *p* < 0.001) and medication literacy (*r* = −0.293, *p* < 0.001). Medication literacy was positively associated with medication adherence (*r* = 0.188, *p* < 0.01). The number of chronic diseases was negatively associated with medication adherence (*r* = −0.172, *p* < 0.05).

**Table 3:** Correlations among stigma, medication adherence, and medication literacy (*n* = 221).

Variable	Self- stigma	Enacted stigma	Medication adherence	Medication literacy	Number of chronic diseases
Stigma	0.972^**^	0.945^**^	−0.259^***^	−0.293^***^	0.123
Self-stigma	1	0.841^**^	−0.162^*^	−0.171^*^	0.130
Enacted stigma	0.841^**^	1	−0.136^*^	−0.208^**^	0.102
Medication adherence	−0.162^*^	−0.136^*^	1	0.188^**^	−0.172^*^
Medication literacy	−0.171^*^	−0.208^**^	0.188^**^	1	0.140^*^

^*^p < 0.05; ^**^p < 0.01, ^***^p < 0.001.

### Mediation analysis among medication literacy, stigma and medication adherence

**Figure 1:**
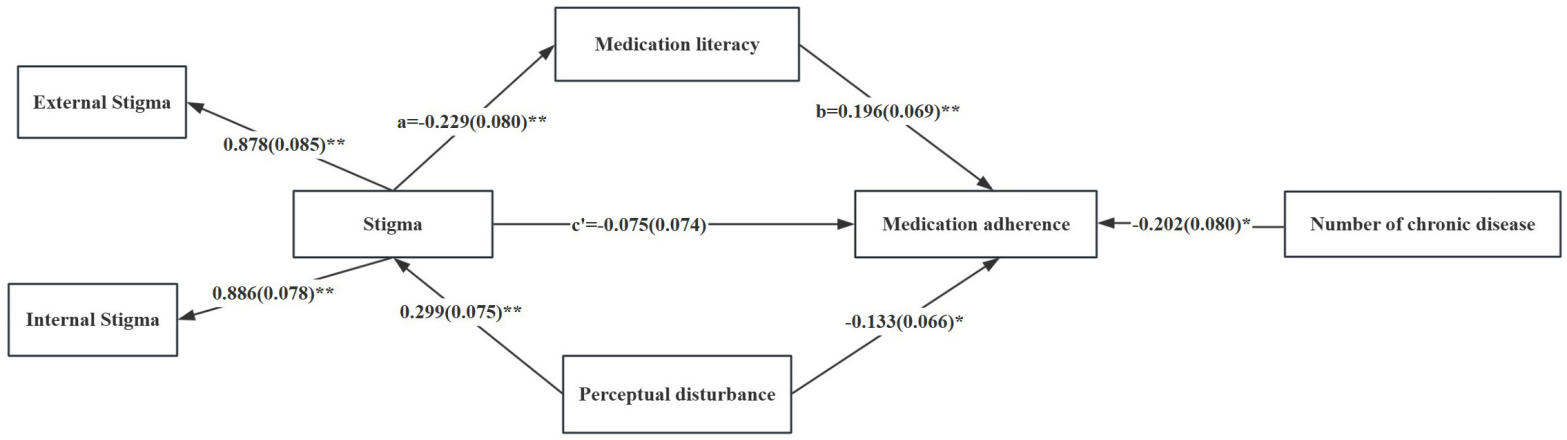
The mediation model. The effect of stigma on medication literacy is a = −0.229 (SE = 0.080, *p* < 0.01); after controlling for stigma, the effect of medication literacy on medication adherence is b = 0.196 (SE = 0.069, *p* < 0.01); the direct effect of stigma on medication adherence is c′ = −0.075 (SE = 0.074, *p* > 0.05). The effects of stigma on enacted stigma and self-stigma are 0.878 (SE = 0.085, *p* < 0.01) and 0.886 (SE = 0.078, *p* < 0.01), respectively. The effect of the number of diseases on medication adherence is −0.202 (SE = 0.080, *p* < 0.05). *p < 0.05, **p < 0.01.

The results of mediation analysis showed that the model fitted well (χ^2^/df = 1.12, CFI = 0.997, TLI = 0.992, RMSEA = 0.024, SRMR = 0.041). In the mediation analysis, medication adherence was treated as the outcome variable and stigma as the explanatory variable. Medication literacy served as a full mediator in the relationship between stigma and medication adherence [β = −0.045, SE = 0.022, 95% CI (−0.095, −0.001); see Figure 1 and Table 4]. The results (Figure 1 and Table 5) revealed that stigma was negatively associated with medication literacy (β = −0.229, *p* < 0.05). Medication literacy was positively associated with medication adherence (β = 0.196, *p* < 0.05). The indirect and direct associations accounted for 37.5 and 62.5% of the total association, respectively (Table 4). Lower stigma levels were associated with higher medication literacy (*r* = −0.293, *p* < 0.001), and higher medication literacy was in turn associated with better medication adherence (*r* = 0.188, *p* < 0.01). Medication literacy mediated the association between stigma and medication adherence.

**Table 4:** Results for effects of stigma on medication adherence with medication literacy as a mediator.

Variable	Effect	BootSE	BootLLCI	BootULCI	Relative effect size (%)
Total effect	−0.120	0.068	−0.257	0.058	100
Direct effect	−0.075	0.074	−0.223	0.105	62.5
Indirect effect	−0.045	0.022	−0.095	−0.001	37.5

**Table 5:** Testing the mediation effect of stigma on medication adherence.

Results variable^*^	Predictive variable^*^	β	SE	*p*-value
Medication literacy	Stigma	−0.229	0.080	0.004
Stigma	Perceptual/sensory disturbance (yes = 1, no = 0)	0.299	0.075	< 0.01
Self-stigma	Stigma	0.886	0.078	< 0.01
Enacted stigma	Stigma	0.878	0.085	< 0.01
Medication adherence	Medication literacy	0.196	0.069	0.005
	Stigma	−0.075	0.074	0.310
	Perceptual/sensory disturbance (yes = 1, no = 0)	−0.133	0.066	0.042
	Number of chronic diseases	−0.202	0.080	0.012

^*^Except perceptual/sensory disturbance, used the original continuous value for all other variables.

Covariates included the number of chronic diseases and perceptual/sensory disturbance severity (both identified as significant predictors of adherence in univariate analyses, *p* < 0.05). Additionally, the number of chronic diseases was negatively associated with medication adherence (β = −0.202, *p* < 0.05). Perceptual/sensory disturbance emerged as a significant predictor of both medication adherence (β = −0.133, *p* < 0.05) and stigma (β = 0.299, *p* < 0.01).

## Discussion

In this study, 221 patients with multimorbidity exhibited moderate levels of medication adherence. Medication nonadherence among this population was reported to be associated with adverse clinical outcomes (e.g., disease progression and preventable hospitalizations) and increased health care expenditures (38). Many factors are related to medication adherence in patients with multimorbidity. Demographic characteristics and disease-related conditions are unmodifiable factors, while psychological factors may represent a key area for improving medication adherence in this population (38). In this study, we examined the association and potential mechanism between stigma and medication adherence in patients with multimorbidity. The results indicated that patients with more chronic conditions (β = −0.202, *p* < 0.05) and greater perceptual/sensory impairment (β = −0.133, *p* < 0.05) had lower medication adherence scores. Stigma was negatively associated with medication adherence and medication literacy, and medication literacy played a full mediating role in the association between stigma and medication adherence.

Patients with multimorbidity often lack correct medication beliefs and hold a compromising attitude toward polypharmacy (39). They may not fully understand the benefits of treatment, regard medication taking as an unavoidable obligation (40), and even develop avoidance tendencies that lead to unauthorized discontinuation (41, 42). Medication literacy was positively associated with medication adherence (β = 0.196, *p* < 0.05). Medication literacy may help patients understand basic medical knowledge and take medications as prescribed (43), whereas stigma may be negatively associated with patients' confidence and willingness to improve medication literacy (42). In this study, medication literacy was found to mediate the association between stigma and medication adherence. Stigma may also be related to higher perceived impairment (e.g., fear of social judgment), which in turn is associated with lower medication adherence (22). However, the direct association between stigma and medication adherence was not statistically significant in the present study, which may be attributed to the relatively low level of stigma in this sample.

Many studies have reported a positive correlation between medication literacy and medication adherence in patients with chronic diseases (39, 40), and stigma has been identified as a factor associated with poorer outcomes (15). A previous study by Waite et al. (3) reported that stigma acted as a mediator between literacy and medication adherence in HIV patients. In that study, literacy primarily referred to reading and writing ability, which is closely linked to educational level. However, medication literacy encompasses not only basic literacy but also the ability to understand fundamental medical information and adhere to medication regimens. To the best of our knowledge, this is the first study to demonstrate that medication literacy mediates the association between stigma and medication adherence in patients with multimorbidity.

Based on the observed associations, enhancing medication literacy may serve as a viable starting point for addressing medication adherence among this population. In our sample, greater knowledge of medication-related information was associated with fewer patient concerns. Pakistani researchers Kamal et al. (44) developed an m-Health-based interactive voice response service, which was demonstrated to improve patients' medication literacy. Similarly, the electronic teaching tool, the “digital drag-and-drop pillbox,” designed by Granger et al. (45), also exhibited positive effects on enhancing medication literacy.

Furthermore, although the overall stigma level was low in this cohort, stigma was indirectly linked to medication adherence through the mediating role of medication literacy. Although no significant direct effect of stigma on medication adherence was observed in the adjusted direct path of the SEM model, a significant negative bivariate correlation was found (*r* = −0.259, *p* < 0.001). This pattern may be explained by the mediation effect of medication literacy as well as potential suppression or confounding effects of covariates included in the model, indicating that stigma may exert an indirect influence on medication adherence through medication literacy. These findings suggest that future research may explore stigma-reduction interventions to potentially facilitate improvements in medication literacy, which could in turn be associated with better medication adherence among patients with multimorbidity.

Additionally, this study further revealed that the number of chronic diseases and the presence of perceptual disturbance are associated with poor medication adherence. A prior study reported that receiving more than four medications per visit was more likely to be associated with nonadherence compared with receiving only one medication (46). Specifically, a higher number of chronic diseases tends to result in more complex treatment regimens, which require more sophisticated cognitive abilities to understand and adhere to prescribed medication schedules. However, age-related declines in hearing, vision, memory, and other sensory-perceptual functions are common among older individuals, which may also impair their ability to recognize and distinguish medications.

Consistent with previous studies (47), patients with perceptual/sensory impairment exhibited higher levels of self-stigma (*r* = −0.276, *p* < 0.05). Perceptual/sensory disturbance also emerged as a significant predictor of both medication adherence (β = −0.133, *p* < 0.05) and stigma (β = 0.299, *p* < 0.01). This dual association underscores the need for tailored interventions that address perceptual disturbance to improve medication adherence while mitigating its potential links to stigma (e.g., sensory-friendly medication reminders, smart pill boxes). In the present study, the mean self-stigma score was 1.15 (1.0, 2.0), which was slightly higher than the mean enacted stigma score of 1.00 (1.00, 1.36). This discrepancy may be attributed to the tendency of patients with multimorbidity to internalize illness-related negative perceptions and experiences, leading to self-blame, guilt, and subsequent self-stigma. Providing enhanced health education and social support to patients with multimorbidity, and assisting them in developing a correct understanding of their conditions, may help alleviate their self-stigma.

This study also has several limitations. Firstly, this study is a cross-sectional study, and it is not possible to observe the dynamic changes in the influence of various variables on medication adherence over time. Second, this study collected data from only patients with multimorbidity by convenience sampling from one tertiary hospital in Beijing, which may limit the external validity and generalizability of our findings. The small sample size and patients from the same environment (same ward) may be one of the reasons for the insignificant direct effects of stigma and medication adherence in this study. Third, for decreasing the patients' questionnaire filling burden, the perceptual/sensory disturbances were only evaluated by the self-reported simple items, the prevalence of the perceptual/sensory disturbances might be underreported. Future studies are recommended to adopt objective measurements or standardized assessment scales to obtain more accurate and reliable evaluations of sensory and perceptual function. Fourth, the present study performed multiple univariate subgroup comparisons without adjustment for multiple comparisons. Thus, the results are exploratory and should be interpreted with caution. Although some stigma differences were statistically significant, their small magnitude suggests limited clinical relevance. The last, unmeasured confounders, such as medication burden, treatment complexity, and socioeconomic barriers, may also influence the observed associations. In the future, multicenter, large-sample, longitudinal studies can be carried out, and qualitative and mixed studies can be conducted to further explore the role of psychosocial factors in medication adherence of patients with multimorbidity.

## Conclusion

Medication literacy exerts a mediating effect between stigma and medication adherence in patients with multimorbidity. As a cross-sectional observational design, this research provides a reference basis for subsequent intervention studies. In the future, targeted psychological intervention and health education research can be carried out to test the intervention effect for improving the medication adherence in patients with multimorbidity.

## Data Availability

The original contributions presented in the study are included in the article/Supplementary material, further inquiries can be directed to the corresponding author/s.
