# Supplementary material for: The impact of stigma on medication adherence among patients with multimorbidity: mediation analysis of medication literacy
Source: Front Public Health. 2026 Apr 30;14:1782369. doi: 10.3389/fpubh.2026.1782369 (PMC13171576; doi:10.3389/fpubh.2026.1782369)
Supplement: Supplementary file 1 [file Table_1.docx]

Table S1. Univariate analysis of medication literacy of patients with multimorbidity (n=221)

| Variable |  | n (%) | Medication literacy | | |
| --- | --- | --- | --- | --- | --- |
|  |  |  | Mean ± SD | F/t | P |
| Age | ≤45 | 15(6.79) | 9.63 ±3.20 | 1.633 | 0.197 |
|  | 46-64 | 72(32.58) | 7.84 ±3.95 |  |  |
|  | ≥65 | 134(60.63) | 8.36 ±3.57 |  |  |
| Gender | Male | 124(56.11) | 8.49 ±3.48 | 0.617 | 0.538 |
|  | Female | 97(43.89) | 8.20 ±3.70 |  |  |
| Level of education | Junior high school and below | 100(45.25) | 8.36 ±3.51 | 1.468 | 0.233 |
|  | High school | 81(36.65) | 7.81 ±3.47 |  |  |
|  | Bachelor and above | 40(18.10) | 8.95±4.03 |  |  |
| Marital status | No spouse | 22(10.0) | 8.81±3.08 | 0.590 | 0.443 |
|  | Having spouse | 199(90.0) | 8.20 ±3.65 |  |  |
| Payment scheme | Medical insurance | 173(78.28) | 8.09 ±3.67 | 3.675 | 0.027* |
|  | Free medical service | 31(14.03) | 9.12 ±3.07 |  |  |
|  | NCMS and Self-paying | 17(7.69) | 6.41±3.92 |  |  |
| Co-resident | With family | 201(90.0) | 8.19±3.60 | 0.634 | 0.427 |
|  | Live alone | 20(9.0) | 8.90±3.57 |  |  |
| Number of chronic diseases | 2-3 | 123(55.66) | 7.76±3.88 | 2.737 | 0.067 |
|  | 4-5 | 72(32.58) | 8.90±3.25 |  |  |
|  | ≥ 6 | 26(11.76) | 8.85±2.81 |  |  |
| Duration of disease (years) | 0-3 | 47(21.27) | 7.77±3.83 | 0.626 | 0.536 |
|  | 4-9 | 44(19.91) | 8.57±3.67 |  |  |
|  | ≥10 | 130(58.82) | 8.33±3.50 |  |  |
| Perceptual/sensory disturbance | Yes | 72(32.58) | 7.91±3.58 | 0.958 | 0.329 |
|  | No | 149(67.42) | 8.42±3.60 |  |  |
| Hospitalizations | First | 79(35.75) | 7.86±3.98 | 1.498 | 0.222 |
|  | Many times | 142(64.25) | 8.47±3.36 |  |  |
| Type of medication | 1-2 | 48(21.72) | 8.51±3.55 | 0.505 | 0.679 |
|  | 3-5 | 93(42.08) | 7.92±3.67 |  |  |
|  | 5-9 | 71(32.13) | 8.40±3.71 |  |  |
|  | ≥10 | 9(4.07) | 9.00±2.23 |  |  |

NCMS: New rural cooperative medical system.

*: Patients with NCMS/Self-paying payment scheme had lower medication literacy than patients in other two groups (*p* <0.05).

Table S2. Univariate analysis of stigma of patients with multimorbidity (n=221)

| Variable |  | n (%) | SSCI | | |
| --- | --- | --- | --- | --- | --- |
|  |  |  | M (IQR) | F/Z | P |
| Age | ≤45 | 15(6.79) | 35(24, 49) | 2.175 | 0.337 |
|  | 46-64 | 72(32.58) | 26.5(24, 42) |  |  |
|  | ≥65 | 134(60.63) | 25(24, 40) |  |  |
| Gender | Male | 124(56.11) | 25(24, 42) | 0.902 | 0.342 |
|  | Female | 97(43.89) | 27(24, 44) |  |  |
| Level of education | Junior high school and below | 100(45.25) | 26(24,45.25) | 4.068 | 0.131 |
|  | High school | 81(36.65) | 27(24, 48) |  |  |
|  | Bachelor and above | 40(18.10) | 25(24,29.5) |  |  |
| Marital status | No spouse | 22(10.0) | 29(24, 73.75) | 4.239 | 0.039 |
|  | Having spouse | 199(90.0) | 26(24, 42) |  |  |
| Provider payment | Medical insurance | 173(78.28) | 26(24, 42.5) | 5.822 | 0.121 |
|  | Free medical service | 31(14.03) | 25(24, 27) |  |  |
|  | NCMS and Self-paying | 17(7.69) | 36(24,60) |  |  |
| Co-resident | With family | 201(91.0) | 26(24, 42.5) | 0.974 | 0.324 |
|  | Live alone | 20(9.0) | 25.5(24, 40) |  |  |
| Number of chronic diseases | 2-3 | 123(55.66) | 26(23, 38) | 4.724 | 0.118 |
|  | 4-5 | 72(32.58) | 24(24, 41.5) |  |  |
|  | ≥ 6 | 26(11.76) | 39.5(24, 69) |  |  |
| Duration of disease（years） | 0-3 | 47(21.27) | 25(24,38) | 0.042 | 0.838 |
|  | 4-9 | 44(19.91) | 25.5(24, 41) |  |  |
|  | ≥10 | 130(58.82) | 27(24, 44.25) |  |  |
| Perceptual/sensory disturbance | Yes | 72(32.58) | 35(24, 64.5) | 12.894 | <0.001 |
|  | No | 149(67.42) | 25(24, 35.75) |  |  |
| Hospitalizations | First | 79(35.75) | 24(24, 35) | 3.963 | 0.047 |
|  | Many times | 142(64.25) | 28(24, 48) |  |  |
| Type of medication | 1-2 | 48(21.72) | 26(24, 36) | 0.185 | 0.980 |
|  | 3-5 | 93(42.08) | 27(24, 43.5) |  |  |
|  | 5-9 | 71(32.13) | 25(24, 44) |  |  |
|  | ≥10 | 9(4.07) | 26(24, 43) |  |  |

NCMS: New rural cooperative medical system.
